# Impedimetric Measurement of Exchange Currents and Ionic Diffusion Coefficients in Individual Pseudocapacitive Nanoparticles

**DOI:** 10.1021/acsmeasuresciau.4c00017

**Published:** 2024-07-11

**Authors:** Brian Roehrich, Lior Sepunaru

**Affiliations:** Department of Chemistry and Biochemistry, University of California Santa Barbara, Santa Barbara, California 93106, United States

**Keywords:** electrochemistry, impedance, fast Fourier transform, single pseudocapacitor, microscopy, SECCM, intercalation

## Abstract

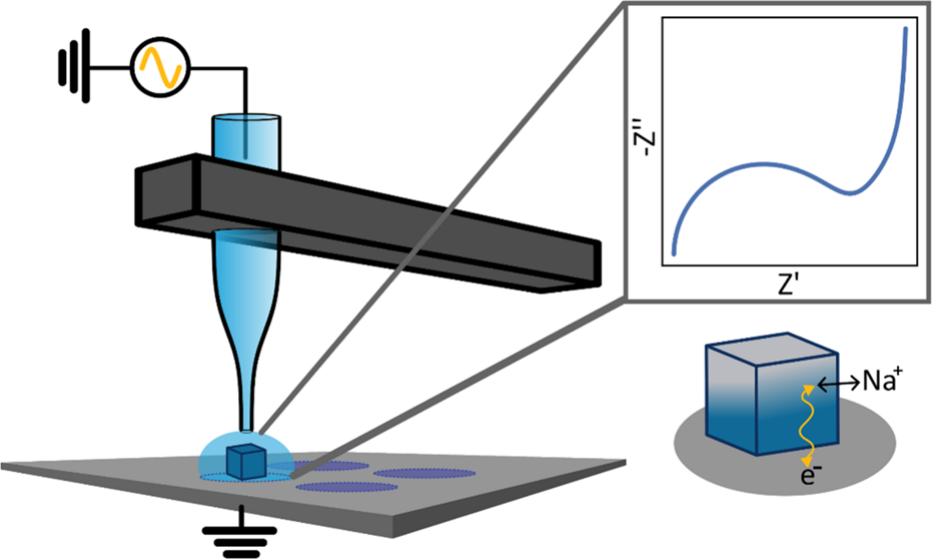

Among electroanalytical techniques, electrochemical impedance spectroscopy (EIS) offers the unique advantage of a high degree of frequency resolution. This enables EIS to readily deconvolute between the capacitive, resistive, and diffusional processes that underlie electrochemical devices. Here, we report the measurement of impedance spectra of individual, pseudocapacitive nanoparticles. We chose Prussian blue as our model system, as it couples an electron-transfer reaction with sodium ion intercalation—processes which, while intrinsically convoluted, can be readily resolved using EIS. We used a scanning electrochemical cell microscope (SECCM) to isolate single Prussian blue particles in a microdroplet and measured their impedance spectra using the multi-sine, fast Fourier transform technique. In doing so, we were able to extract the exchange current density and sodium ion diffusivity for each particle, which respectively inform on their electronic and ionic conductivities. Surprisingly, these parameters vary by over an order of magnitude between particles and are not correlated to particle size nor to each other. The implication of this apparent heterogeneity is that in a hypothetical battery cathode, one active particle may transfer electrons 10 times faster than its neighbor; another may suffer from sluggish sodium ion transport and have restricted charging rate capabilities compared to a better-performing particle elsewhere in the same electrode. Our results inform on this intrinsic heterogeneity while demonstrating the utility of EIS in future single-particle studies.

## Introduction

Nanoparticles are ubiquitous in electrochemistry, but understanding their intrinsic properties is difficult using conventional electroanalytical techniques. This is because the properties of individual electrochemically active particles of the same material may differ greatly from one another due to morphology, crystallinity, composition, or other factors.^1,2^ Typical bulk characterization techniques mask these differences and provide an ensemble-averaged response. Instead, single-entity electrochemistry studies particles one by one—linking their individual properties to that of the ensemble in a bottom-up approach to further improve the material’s properties.^3−7^ Motivated by this, several groups have begun applying the scanning electrochemical cell microscope (SECCM) toward the study of individual electroactive particles.^8−11^ In SECCM measurements, a nano- or micropipette is filled with electrolyte, equipped with a counter electrode, and positioned above a conductive substrate which serves as the working electrode.^8,12^ A bias is applied between the two electrodes, and the pipette is slowly lowered toward the substrate by a piezoelectric positioner until the electrolyte wets the substrate and current flows across the interface. The droplet formed between the tip of the pipette and the substrate comprises a microscopic electrochemical cell which, when interrogated electrochemically using a low-noise amplifier,^13,14^ yields a response that is unique to that particular region of the substrate.^15^ For single-particle studies, nanoparticles are dispersed on the conductive substrate prior to the SECCM experiment. When the droplet contains an electroactive nanoparticle, the thermodynamic and kinetic properties of the reaction occurring at that particle can be measured. Because of this simple and elegant mode of operation, SECCM has been applied to study a wide range of electroactive materials, including the activity of various electrocatalysts,^2,16−18^ charge storage in individual pseudocapacitors,^19,20^ and the intercalation of ions in battery active materials.^21−23^ Its powerful compatibility with complementary microstructure imaging and characterization techniques, which measure topography, morphology, or composition, can offer unprecedented insight into structure–property relationships at the nanoscale.^17,24−28^

To date, the vast majority of SECCM experiments have relied on either amperometry (measuring current at a constant potential) or voltammetry (measuring current as the potential is swept) for their electrochemical analysis. In both techniques, however, the measured current is inherently a convolution of several independent processes—including electron transfer, double-layer capacitance, and mass transport, among others—and separating their contributions is challenging. Electrochemical impedance spectroscopy (EIS), in contrast, readily deconvolutes between these phenomena based on their relative time scales.^29−31^ The advantages of EIS are particularly pronounced in systems with a strong coupling between electronic and ionic conductivity, such as those found in batteries and supercapacitors, due to its ability to decouple the relative rates of electron and ion transport.^32^ In EIS, the electrochemical cell is perturbed by a small-amplitude, sinusoidal (alternating current, AC) voltage. The impedance (*Z*) of the cell is intimately related to the frequency of the AC sine wave—at high frequencies, *Z* is dictated by “fast” processes such as double layer formation and rapid electron-transfer reactions, while at low frequencies, *Z* is determined by the rates of sluggish mass transport or pseudocapacitive intercalation.

Here, we demonstrate the measurement of impedance spectra of individual pseudocapacitive nanoparticles. We chose Prussian blue (PB) as a model system—while first reported as a dye in the early 1700s,^33^ Prussian blue (and its derivatives) has attracted recent interest as a low-cost material for sodium- and potassium-ion battery cathodes due to its coupling of Fe^II/III^ redox with alkali metal ion intercalation.^34−36^ The kinetics of the redox reaction (i.e., the exchange current) and the rate at which ions diffuse through the PB lattice are both critically important to the energy storage performance of the material yet are not well understood at the nanoscale. In particular, and despite extensive work over the past two decades, significant debate remains in the literature over the diffusion coefficient of sodium ions within the PB lattice—reported values range over a staggering 7 orders of magnitude.^37^ To explore this, we measured the impedance spectra of isolated Prussian blue nanocubes using a multi-sine, fast Fourier transform (FFT) technique.^38^ This technique enables rapid measurement (within seconds) of the impedance spectrum, mitigating potential thermodynamic and mechanical drifts that could arise during the SECCM measurement process. We show that the impedance spectrum of a single Prussian blue nanoparticle deconvolutes its electron-transfer reaction and ion mobility kinetics. By recording the spectra of 16 independent particles in the SECCM configuration, we can evaluate the intrinsic heterogeneity in the exchange current and ion diffusivity and show that these rates can vary by an order of magnitude, even among particles synthesized in the same batch.

## Results and Discussion

**Figure 1:**
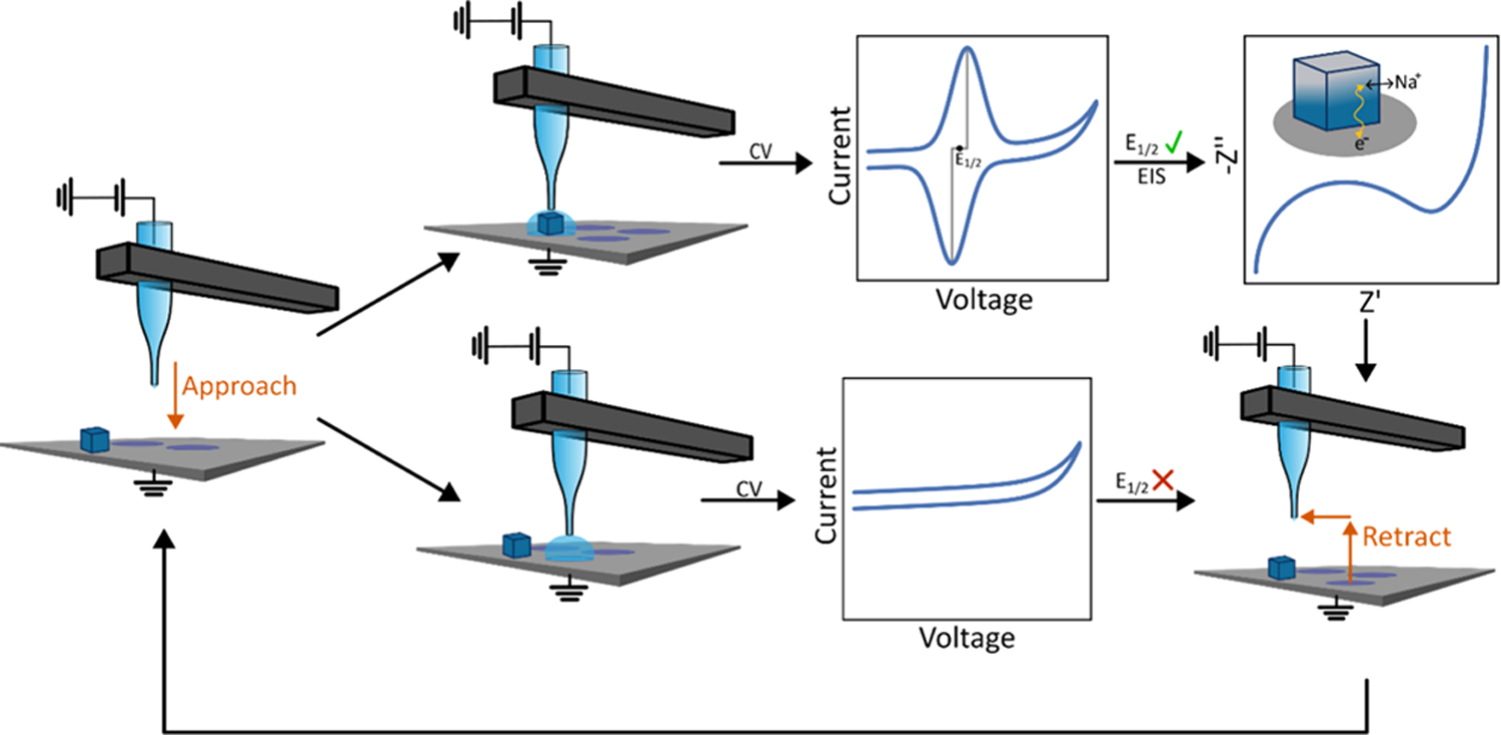
Steps required for single-particle FFT-EIS measurements. The SECCM probe is moved toward the surface until its electrolyte wets the surface. After contact, a cyclic voltammogram is recorded and analyzed to determine if it contains a pair of oxidation and reduction waves. If it does, *E*_1/2_ is determined as the midpoint between the two peak potentials and applied as the DC bias for a subsequent EIS measurement. After acquiring the impedance spectrum (or if no peaks were detected in the CV), the probe is retracted and moved to the next location.

Prussian blue nanocubes were synthesized via the hydrothermal method^39^ and drop-cast on a glassy carbon (GC) substrate to form a highly diluted surface layer. The morphology of the particles and their dispersion on GC were characterized by scanning electron microscopy (SEM) (Figure S1). We performed SECCM using borosilicate glass pipettes, which were pulled to a tip diameter of 3–5 μm (Figure S2), filled with 0.1 M NaCl, and fitted with an Ag/AgCl quasi-reference counter electrode (the potentials of the Ag/AgCl wires in 0.1 M NaCl were typically +44 mV vs saturated calomel electrode (SCE)). The general steps performed in each SECCM experiment are shown in Figure 1. At a series of predefined locations above the substrate, the micropipette was slowly lowered by a piezoelectric positioner until the droplet contacted the surface.^40^ Movement was immediately halted, and then a cyclic voltammogram was recorded and automatically analyzed by the controlling Python program. If the program determined that a PB nanoparticle was present (based on the presence of reversible redox waves, vide infra), an impedance spectrum was recorded before the pipette was retracted and moved to the next location.

**Figure 2:**
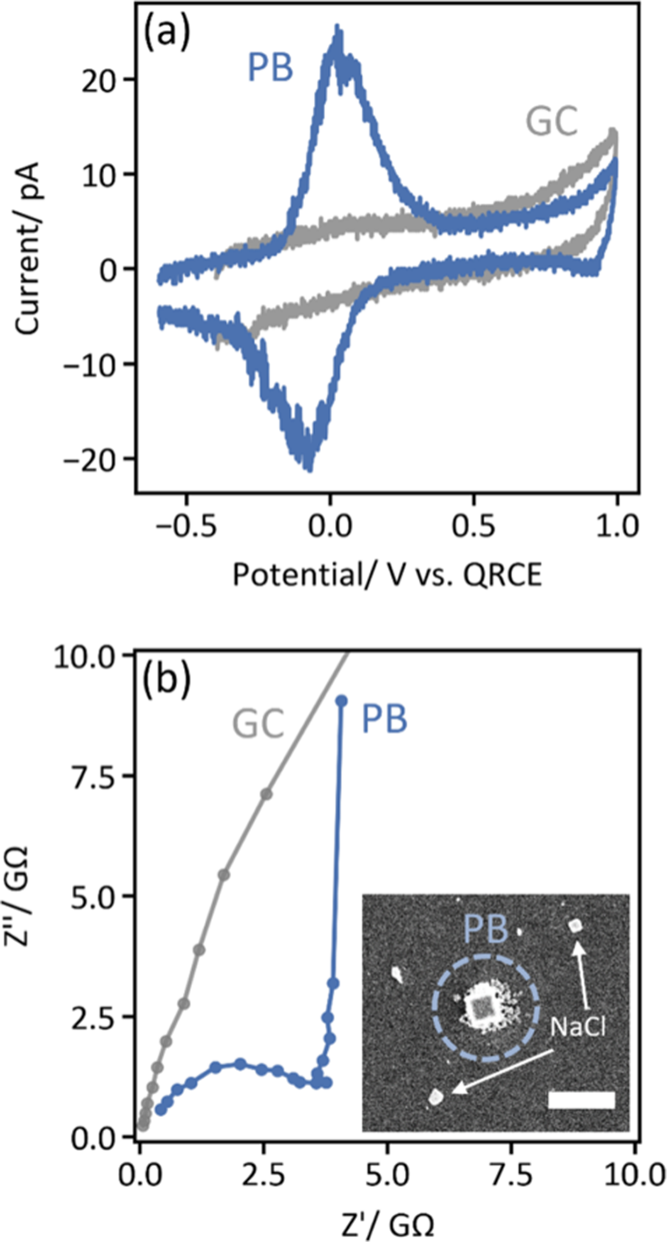
The impedance spectrum of a single Prussian blue nanoparticle is clearly distinguishable from the background. (a) Cyclic voltammograms (1 V/s) recorded when the SECCM probe was positioned over the bare glassy carbon substrate (“GC”, gray) and when the droplet encompassed a Prussian blue nanoparticle (“PB”, blue). The redox waves visible for the PB NP are attributed to the Fe^II^Fe^II^/Fe^II^Fe^III^ couple, i.e., the transition between Prussian white and Prussian blue. (b) Impedance spectra recorded using the FFT technique at the same locations. *E*_1/2_ (−20 mV vs the Ag/AgCl QRCE for this particle) was applied as the DC bias for both EIS experiments, and the impedance was measured at 18 frequencies between 1 Hz and 1 kHz with an AC amplitude of 50 mV_pp_ (Figure S3). The impedance magnitudes recorded on the PB NP fall in the range of GΩ yet are still significantly smaller than those recorded on the bare substrate at the same frequencies. Inset: an SEM image of the same PB NP. Salt deposits left behind by nearby SECCM hopping points are indicated. The scale bar is 2 μm.

Cyclic voltammograms obtained when the SECCM probe was positioned over an individual PB nanocube contained characteristic peaks associated with quasi-reversible, surface-bound, redox activity. The SECCM held the substrate at −600 mV for 5 s after contact was established. If the PB NP was present, this step reduced the particle into the Fe^II^–Fe^II^ (Prussian white) state. Then, the potential of the glassy carbon substrate was swept to +1 V vs Ag/AgCl and back at a scan rate of 1 V/s (the high scan rate was chosen to minimize the total time of the SECCM experiment). No redox features were observed if the micropipette was in contact with the bare glassy carbon substrate (Figure 2a, gray), while a pair of quasi-reversible waves was visible if the droplet contained a PB nanoparticle (Figure 2a, blue). These peaks correspond to the one-electron oxidation of the particle to the Fe^II^–Fe^III^ (Prussian blue) state. The half-wave potential of this reaction (for this particle, −20 mV vs Ag/AgCl|0.1 M NaCl or 30 mV vs SCE) is similar to that previously reported for sodium-containing Prussian blue.^41^ Furthermore, the symmetric shape of the waves is as expected for quasi-reversible, surface-bound electrochemistry.^42,43^

The impedance spectrum of the same nanoparticle contains features that deconvolute the electronic and ionic components that underlie the overall electrochemical reaction. EIS was performed using a multi-sine waveform, which contained 18 frequencies spanning 1 Hz to 1 kHz. We chose to use the multi-sine, fast Fourier transform technique to minimize the acquisition time of each spectrum—a full spectrum, averaged over 5 cycles of the lowest frequency, was measured in 5 s.^38,44,45^ The waveform (Figure S3) was normalized to have an amplitude of 50 mV_pp_ and applied with a DC bias set as *E*_1/2_ from the NP’s cyclic voltammogram. The measured impedance spectrum, when represented as a Nyquist plot, contains a semicircle in the high-frequency regime while trending toward a large imaginary impedance in the low-frequency limit (Figure 2b). These features are characteristic of an ion-intercalating material and qualitatively match those observed in bulk PB films.^46^ Because the reduction/oxidation reactions occur at iron centers within the Prussian blue crystal lattice, electron transfer must be accompanied by (sodium) ion intercalation to maintain charge neutrality. At high frequencies, the current response (and thus the measured impedance) is limited by electron transport to surface and near-surface iron centers to which ion transport is facile. At lower frequencies, sodium ions have more time to diffuse further into the crystal lattice, and the impedance is dictated by their transport. At all but the highest frequencies we examined (where solution resistance dominates), the impedance measured in the presence of the PB NP (Figure 2b, blue) is much lower than that measured in its absence (Figure 2b, gray). As current flows through the path of least impedance, this means the contribution of the background to the measured impedance spectrum is small.

**Figure 3:**
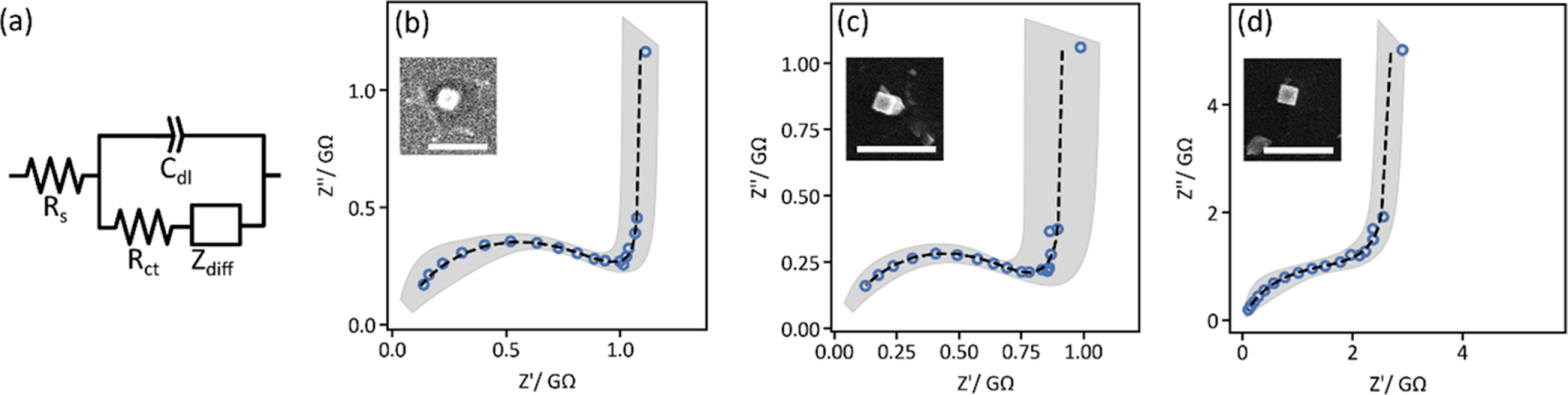
Impedance spectra measured from individual Prussian blue nanocubes are described well by a simple equivalent circuit model. (a) Equivalent electronic circuit used for fitting impedance spectra, with resistors accounting for the solution (*R*_s_) and charge-transfer (*R*_ct_) resistances, a constant phase element representing the double-layer capacitance (*C*_dl_), and a diffusion element modeling the transport of sodium ions within the nanocube (*Z*_diff_).^47^ (b–d) EIS data (open circles) and equivalent circuit fits (dotted curves, shaded regions represent 95% confidence intervals) for three representative PB nanoparticles. Insets are SEM images of each particle that produced the respective impedance spectrum (scale bars are 2 μm).

We used equivalent circuit modeling to extract relevant physical parameters from each individual Prussian blue nanoparticle. The simple, four-element equivalent circuit (Figure 3a) accounts for the bulk solution resistance (*R*_s_), the double-layer capacitance (C_dl_), the charge-transfer resistance between the glassy carbon electrode and the PB NP (*R*_ct_), and diffusion of sodium ions within the PB NP (*Z*_diff_). The double-layer capacitance was modeled as a constant phase element because it includes contributions from both the nanoparticle and the glassy substrate support—their capacitances, while different, are parallel and thus indistinguishable pathways for current to flow. The value of α, which dictates the phase of the constant phase element, was typically ∼0.8 due to these bifurcated capacitive pathways (an ideal capacitor has α = 1). Meanwhile, to approximate the diffusion and intercalation of ions in our cubic particles, we adopted a finite-space Warburg element model for a spherical particle, which accounted for the complementary effects of ion diffusion and pseudocapacitive intercalation.^47,48^ The net equivalent circuit (Figure 3a) was able to model each impedance spectrum we measured from 16 individual PB nanoparticles (three representative particles are shown in Figure 3b–d; the full data set is shown in Figures S4 and S5 and Table S1).

Based on the charge-transfer resistance (*R*_ct_) and diffusional impedance (*Z*_diff_) obtained from each particle’s impedance spectrum, we were able to estimate the exchange current density (*j*_0_) and ionic diffusion coefficient (*D*_Na_). The exchange current density, *j*_0_, was calculated from the fitted value of *R*_ct_ and the contact area (*A*_contact_, estimated by SEM) between the individual nanoparticle and the carbon substrate
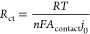
1where *R* is the gas constant, *T* is temperature, *n* is the number of electrons (1), and *F* is Faraday’s constant. We found that *j*_0_ fell in the range of ∼10–200 A m^–2^. As a point of comparison, these values are on the order of the highest reported exchange current densities for lithium-ion cathode materials.^49^ The high *j*_0_ measured for PB may be due to its relatively higher conductivity and enhanced electrochemical reversibility. Likewise, the diffusional impedance was parametrized into an effective resistance *R*_d_ and an intercalation capacitance *C*_d_ (which is strongly correlated with the charge passed in the voltammogram, as shown in Figure S6), yielding a time constant τ from which the diffusion coefficient can be calculated^50,51^
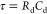
2
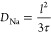
3where *l* is the characteristic diffusion length. In this case, we set *l* = 20 nm for all particles regardless of particle size due to several recent reports showing that the diffusion length in Prussian blue particles is likely on the order of tens of nanometers.^37,52^ We note that choosing a different value of *l* would shift the distribution of diffusion coefficients to higher or lower values without changing their dispersity, as shown by eq 3. Indeed, inconsistent choices of *l* are likely a major reason for the wide range of diffusion coefficients reported in the literature. With this choice of *l*, the values of *D*_Na_ we obtained (∼10^–13^ – 10^–15^ m^2^ s^–1^) fall well within the broad range of previously reported values.^37^

**Figure 4:**
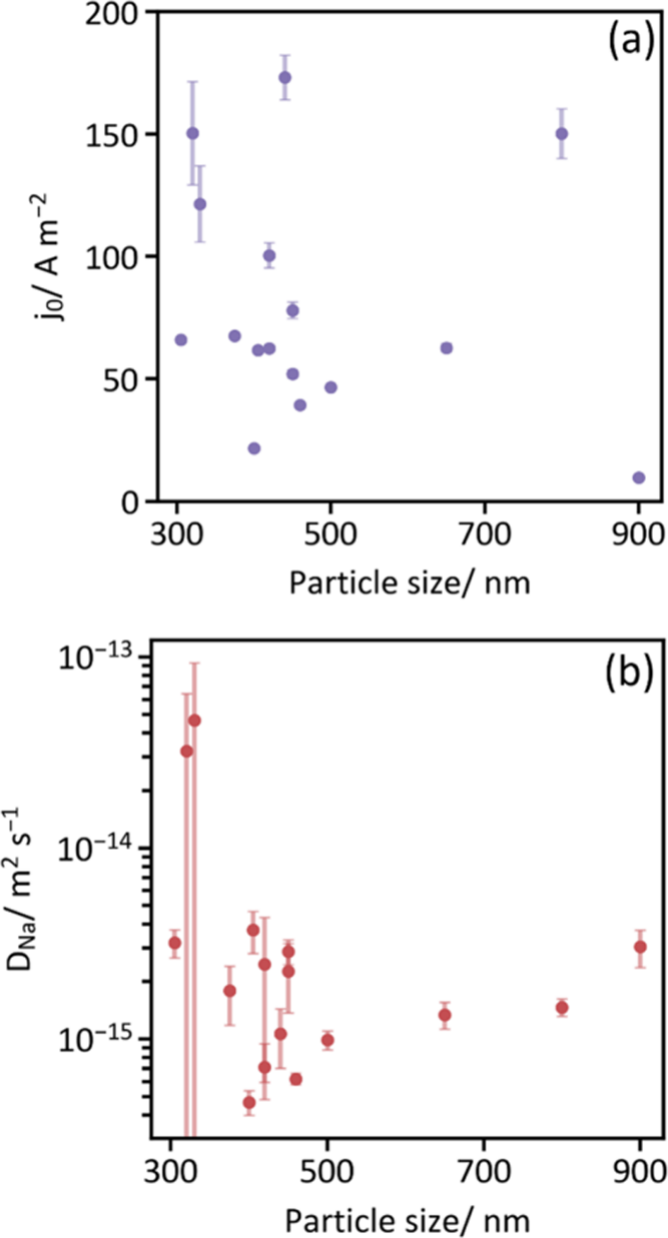
Exchange current densities and sodium ion diffusivities vary by more than an order of magnitude between particles but are not correlated to particle size. (a) Exchange current density (*j*_0_, normalized to particle–substrate contact area, estimated by SEM) and (b) diffusion coefficient (*D*_Na_) as a function of particle size. Here, particle size refers to the average of the two side lengths estimated by SEM. Error bars represent the uncertainty associated with the equivalent circuit fit.

By comparing the exchange current densities (Figure 4a) and solid-state diffusion coefficients (Figure 4b) obtained from 16 individual nanocubes, we can begin to assess the inherent heterogeneity in these parameters across the material. Surprisingly, although all Prussian blue particles originated from the same synthetic batch, *j*_0_ and *D*_Na_ vary by factors of 20 and 10 (excluding the two outlier points whose error bars overlap with all others), respectively. This implies that in a hypothetical battery cathode, one active PB particle can undergo reversible electrochemistry up to 20 times faster than its neighbor, or may transport sodium ions 10 times slower than a better-performing particle elsewhere in the electrode. Evidently, neither *j*_0_ nor *D*_Na_ trend with particle size (Figure 4), nor are they strongly correlated to each other (Figure S7). Interestingly, this phenomenon mirrors a recent observation in individual mesoporous NMC522 particles by Min et al., who found no correlation between either electron transfer or diffusion time scales with secondary particle size.^53^ The authors suggested the particle-to-particle variability observed was due to either different degrees of electrolyte penetration within the secondary particle or inherent heterogeneity between the primary particles.^53^ However, the argument of electrolyte permeation does not explain the variability we observe here. The Prussian blue nanocubes are primary particles that have little electrolyte permeation; thus, our results suggest such variability is inherent. While further work is needed to understand the physical origin of these differences, significant improvements can be made in the active material if particles with high exchange currents and ionic diffusivities can be targeted synthetically.

We performed several experiments to verify the stability and linearity of our measurements. We first tested the linearity of our spectra by measuring sequential impedance spectra with AC amplitudes increasing from 10–200 mV_pp_ on two individual particles (Figure S8). For both particles, the spectra overlap regardless of AC amplitude and do not contain significant distortions at high amplitudes, verifying that the spectra measured herein at 50 mV_pp_ are due to a linear current–voltage relationship. To verify the stability of our impedance spectra, we took sequential, time-resolved measurements of single particles. So long as the particle was in stable electrical contact with the support, the measured impedance spectra did not significantly drift over a 1 min time span (Figure S9). While collecting our data set, we measured several particles which did, however, display significant drift—an example of a particle with poor electrical contact and a nonstationary impedance is shown in Figure S10. The highly fluctuating values of *R*_ct_ observed in that case suggest that the measured resistance is influenced not only by the electrochemical rate constant as discussed above but also by a contact resistance between the particle and the glassy carbon substrate. Seemingly, the contact and charge-transfer resistances are in series with one another and cannot be readily deconvoluted, meaning that differing particle–electrode contact qualities may contribute to the heterogeneity observed in j_0_. Indeed, between 30–50% of the particles we isolated produced large impedances which overlapped with the background due to very poor contact (Figure S11), as has been previously observed.^54^ Still, the time-varying values of *D*_Na_ were constant within error, meaning that their heterogeneity seen in Figure 4B is not affected by the contact resistance but is rather intrinsic to the particles themselves. While the contact resistance remains an open question, these results, taken together, validate the stationarity and linearity of our EIS data and support their physical interpretability.

## Conclusions

In conclusion, we have demonstrated the rapid measurement of the impedance spectra of individual, isolated, pseudocapacitive Prussian blue nanoparticles using SECCM. Despite the impedance falling in the range of GΩ, the impedance spectrum of a single particle is easily distinguishable from the background, and the spectra are stationary and linear when measured using the multi-sine, FFT method. Single-particle spectra are well described by an equivalent circuit incorporating both electronic conductivity and ion transport within the particle, yielding values for the exchange current density and ionic diffusivity for each nanoparticle. We found that these parameters can vary by an order of magnitude, even among particles from the same synthetic batch. Because these variations could not be clearly linked to the size or morphology of the particles, future work is needed to closely examine the nanostructure of individual particles and uncover the origin of these heterogeneities. For example, performing electron diffraction on single particles in transmission electron microscopy (TEM) imaging may be able to quantify the number of iron vacancies, which has been linked to PB capacity and charging performance.^39^

We foresee EIS coupled to SECCM as a valuable tool for future single-particle studies. It should prove more generally applicable than related optical measurements of single nanoparticle impedance,^52,55,56^ which rely on the material’s optical properties changing with voltage—a material-specific phenomenon which may be small or nonexistent for some materials. Our method, which uses current as a direct measurement of the impedance, is universal to any electroactive material. Meanwhile, the range of spatial resolutions possible in SECCM will enable measurements on regions of particles, primary particles, and small ensembles of particles to complement established secondary-particle microscale measurements.^1,53,57,58^ When combined with the high-throughput nature of SECCM (particularly if combined with “smart” probe positioning to target isolated nanoparticles),^22,59^ these benefits will enable rapid screening of structure–property relationships at the single-particle level for batteries, pseudocapacitors, and electrocatalysts.

## Experimental Section

### Preparation of Prussian Blue Nanocubes

All materials were used as received without further purification. Sodium ferrocyanide decahydrate (0.972 g, Acros Organics) was dissolved in 100 mL of Milli-Q water. Two millilitres 37% HCl (Fisher) was added, and the solution was stirred at 60 °C for 4 h.^39^ The deep blue precipitate was recovered by filtration, washed with water and ethanol for 3 times each, and then dried in a vacuum oven at 60 °C overnight.

To prepare samples for SECCM analysis, NPs were suspended at 0.1 mg/mL in water. The suspension was dispersed using a high-power tip sonicator for ∼20 s and then diluted by a factor of 3 with isopropanol. 10 μL of this solution was dropped onto a clean glassy carbon substrate (Ted Pella) and allowed to dry at 50 °C. Glassy carbon substrates were prepared by polishing sequentially on 1, 0.3, and 0.05 μm alumina and then on a clean, wet polishing pad before sonication in isopropanol and water.

### Pipette Fabrication

Pipettes were fabricated from filamented borosilicate capillaries (BF120–94–15, Sutter Instruments) using a Sutter P-2000. The following parameters were used to pull pipettes of approximately 3 μm tip diameter: HEAT 350 FIL 3 VEL 40 DEL 220 PULL 0. The radii of several representative pipettes were confirmed using SEM (Figure S2).

Ag/AgCl wires were created by soldering a short length of silver wire (0.005″, 99.9%, Thermo Scientific) to a gold connector pin. Wires were soaked overnight in household bleach (Clorox, 3.5%) to form an AgCl coating and then rinsed with water. The wires’ potentials were measured in 100 mM NaCl and found to be 43.76 ± 0.54 mV vs saturated calomel electrode (SCE, error represents the standard deviation between four independent wires). Immediately prior to each SECCM experiment, a pipette was filled with an electrolyte (100 mM NaCl) using a MicroFil needle. An Ag/AgCl wire was inserted and secured in place using heat-shrink tubing, which also served to minimize electrolyte evaporation from the back of the pipette.

### Scanning Electrochemical Cell Microscope

SECCM was performed using a home-built instrument that ran using a custom Python program (available at https://github.com/SepLabUCSB/SECM). The substrate of interest was placed on an *XYZ* microscope stage, which was equipped with coarse piezoelectric positioners on the *Z*-axis (for rough positioning of the stage while approaching the pipette) and on the Y-axis (for moving the probe to new locations on the substrate). The pipette was mounted above the stage and connected to a closed-loop *XYZ* piezoelectric positioner (Newport XYZ100SG) for fine control. During experiments, the substrate and pipette tip were enclosed in a plastic container fabricated from the conical end of a 50 mL centrifuge tube. Humidified argon was flowed into an inlet in the container (and out through the top) to maintain a humid environment near the pipette tip and minimize droplet evaporation.^15^

All electrochemical measurements were performed using a HEKA EPC-10 USB. The Ag/AgCl quasi-reference counter electrode within the pipette was grounded, and the (glassy carbon) substrate served as the working electrode. In an SECCM experiment, the GC substrate was mounted to an SEM stub using copper tape and biased at −600 mV. As in electrophysiological patch-clamp experiments, some stray capacitance (and resistance) associated with the pipette and amplifier is present in our system. We used the EPC-10 “C-fast” function to compensate this “fast” capacitance, which typically had a time constant of less than 1 μs. The function was run after the probe was mounted but prior to beginning the approach toward the substrate. At each voltage step in subsequent experiments, the potentiostat uses a compensation circuit to inject a small current opposing that which would flow through the stray capacitance, effectively eliminating this capacitance from measurements. We recorded C-fast when the probe was not in contact with the surface to ensure the compensation did not eliminate the double layer or electrochemical capacitance of the surface. The probe was moved toward the substrate in 10 nm steps (equivalently, ∼0.8 μm/s), the current was recorded at each step, and probe movement was halted as soon as the current magnitude rose above a preset threshold (typically 8 pA), which indicated the droplet had wet the surface. Then, cyclic voltammetry was performed at a scan rate of 1 V/s and analyzed in real time by the controlling Python program. The program searched for oxidation and reduction peaks with prominences greater than 5 pA. If both oxidation and reduction peaks were detected (suggesting the presence of a PB particle), the half-wave potential of the peaks was calculated and applied as a DC bias, and an impedance spectrum was recorded using the Fourier transform technique (vide infra). Then, the pipette was retracted from the surface by 5 μm and moved above the next point. Typically, a 16 × 16 grid of points spanning 75 μm × 75 μm was acquired in a single experiment. The stage was subsequently automatically moved using the coarse *Y*-axis piezoelectric motor to acquire a new grid of data points in a new location. After a series of SECCM experiments, the GC substrate was transferred to a scanning electron microscope (SEM, Thermo Fisher Apreo C) for imaging.

### Fast Fourier Transform Impedance Spectroscopy

Impedance spectroscopy was performed using the FFT technique introduced by Popkirov and Schnidler.^38^ Briefly, an AC waveform containing 18 frequencies of interest (spanning 1 Hz to 1 kHz) was generated by summing together sine waves at each frequency
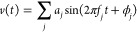
4where *a*_*j*_ is the amplitude and ϕ_*j*_ is the phase at each frequency *f*_*j*_. While phases were randomized, each sine wave’s amplitude was optimized to maximize signal-to-noise.^44^ The waveform (Figure S3) was scaled to have a peak-to-peak amplitude of 50 mV in the time domain. While this amplitude is larger than those most commonly employed in EIS, we chose it to maximize signal-to-noise. We note that the nonuniform power spectrum of the waveform (much smaller amplitudes are applied at higher frequencies, as shown in Figure S3) means that the true voltage perturbation at most frequencies is much smaller than 50 mV. In fact, no single frequency of the measurement is perturbed at the nominal 50 mV amplitude, as the peak-to-peak amplitude of the summed waveform is caused by constructive interference of multiple sine waves rather than being determined by a single sine wave. This waveform was added to a DC bias (determined as the midpoint between the oxidation and reduction peaks detected in a particle’s cyclic voltammogram) and filtered at 100 kHz by a 2-pole Bessel filter. The voltage and current (filtered by sequential 10 and 5 kHz 6-pole Bessel filters) were recorded for 5 s and Fourier transformed to obtain an impedance spectrum that averaged over 5 complete cycles of the lowest frequency, 1 Hz. The low-pass filters we applied caused a small phase shift at the highest measured frequencies, which we corrected for by calibrating against the spectrum of a known 10 MΩ resistor, as we previously described.^45^ Impedance spectra were fit using MEISP software (Kumho Petrochemical, Ltd.). We represent all impedance spectra as Nyquist plots, where *Z*′ is the real component and *Z*″ is the negative of the imaginary component of the impedance. 95% confidence intervals of fits were estimated by Monte Carlo simulations (*N* = 10,000) of the parameter space in the range *p* ± 2 × error*_p_*, where *p* is a best-fit value of a parameter (*R*_s_, *R*_ct_, etc.) and error_*p*_ is the uncertainty associated with that parameter.

## Supporting Information Available

Additional characterization of PB nanoparticles by SEM and energy dispersive spectroscopy (EDS); SEM images of micropipettes, additional details on the impedance waveform; EIS data from 16 individual particles, and time-resolved impedance measurements (PDF)
