# Supplementary material for: Impedimetric Measurement of Exchange Currents and Ionic Diffusion Coefficients in Individual Pseudocapacitive Nanoparticles
Source: ACS Meas Sci Au. 2024 Jul 11;4(4):467–74. doi: 10.1021/acsmeasuresciau.4c00017 (PMC11342456; doi:10.1021/acsmeasuresciau.4c00017)
Supplement: Supplementary file 1 — tg4c00017_si_001.pdf [file tg4c00017_si_001.pdf]

Supporting Information: Impedimetric Measurement of Exchange Currents and Ionic Diffusion Coefficients in Individual Pseudocapacitive Nanoparticles

Brian Roehrich[1] and Lior Sepunaru[1]*

Department of Chemistry and Biochemistry, University of California Santa Barbara, Santa Barbara, CA 93106, USA.

*Corresponding author. Email: sepunaru@ucsb.edu

Table of Contents

**3**	PB Particle Identified by Elemental Mapping	Figure S1
**4**	Characterization of Micropipettes	Figure S2
**5**	Waveform Employed for EIS Measurements	Figure S3
**6**	Fitted Impedance Spectra from 16 Nanoparticles	Figure S4
**7**	Fitted Equivalent Circuit Parameters	Table S1
**8**	Equivalent Circuit Elements versus Particle Size	Figure S5
**9**	Correlation Coefficients	Figure S6
**10**	Correlation between D_{Na} and j_0	Figure S7
**11**	Impedance Spectra at Varying AC Amplitudes	Figure S8
**12**	Time-Resolved Impedance is Drift Free	Figure S9
**13**	Example Particle with Fluctuating Conductivity	Figure S10
**14**	Impedance Spectrum with Poor Electrical Contact	Figure S11

Characterization of Prussian Blue Nanocubes

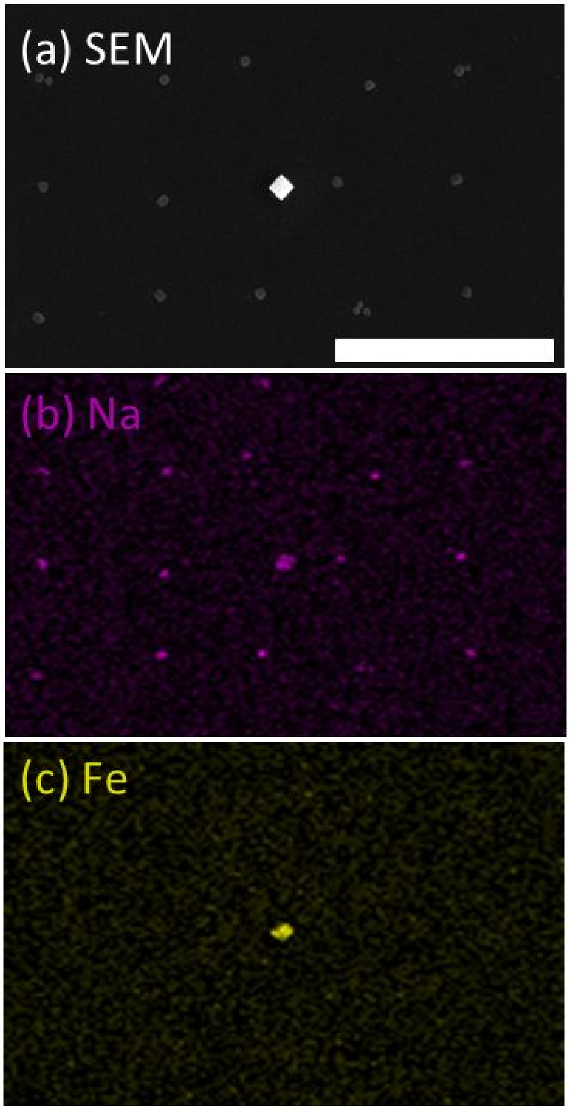

Figure S1. Energy-dispersive X-ray spectroscopy (EDS) distinguishes between Prussian blue nanoparticles and the grid of NaCl deposits left behind by SECCM experiments. Images were acquired using a ThermoFisher Apreo C scanning electron microscope operating at 10 kV with a current of 3.2 nA. (a) Under these imaging conditions the PB particle has high contrast compared to the (carbon) background and the NaCl deposits in the secondary electron micrograph. (b) Spatially-resolved EDS confirms the presence of sodium across the grid of locations probed in the SECCM experiment. A large sodium signal is also seen at the particle's location due to the sodium intercalated in its crystal lattice. (c) Iron is only detected at the particle, distinguishing it from similarly-sized salt deposits.

Characterization of Micropipettes

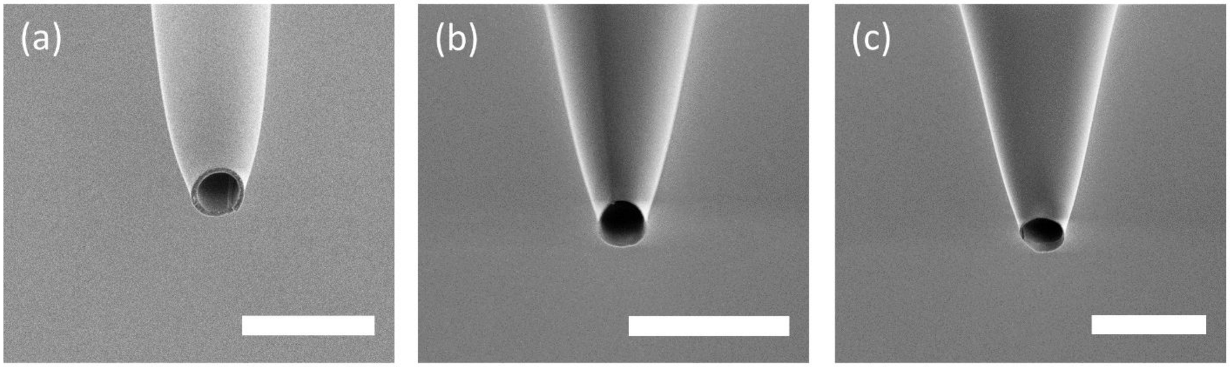

Figure S2. (a-c) Scanning electron micrograph of three representative micropipettes. Scale bars represent 10 µm. The pipette was pulled from 1.2 mm O.D., 0.94 mm I.D., filamented borosilicate glass (BF120-94-15, Sutter) using a P-2000 pipette puller. The pull parameters were HEAT 350 FIL 3 VEL 40 DEL 220 PULL 0.

Waveform Employed for EIS Measurements

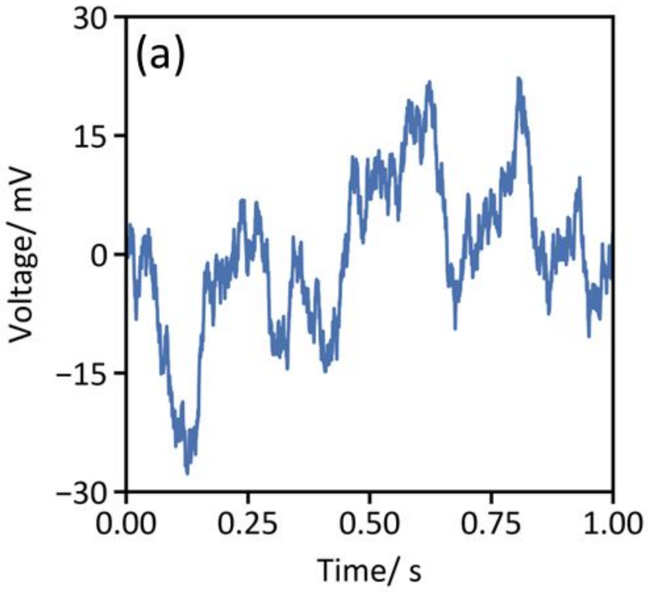

Figure S3. Representations of the waveform used for FFT-EIS measurements in the (a) time and (b) frequency domains. The waveform was scaled to have a 50 mV peak-to-peak amplitude in the time domain. The perturbation amplitude was a function of frequency in order to maximize S/N, as the electrochemical cell has higher impedance at low frequencies. Thus, a larger voltage at low frequency and a smaller voltage at high frequency are necessary to make the current output similar at all frequencies.[1] The phase at each frequency was randomized.

Fitted Impedance Spectra from 16 Nanoparticles

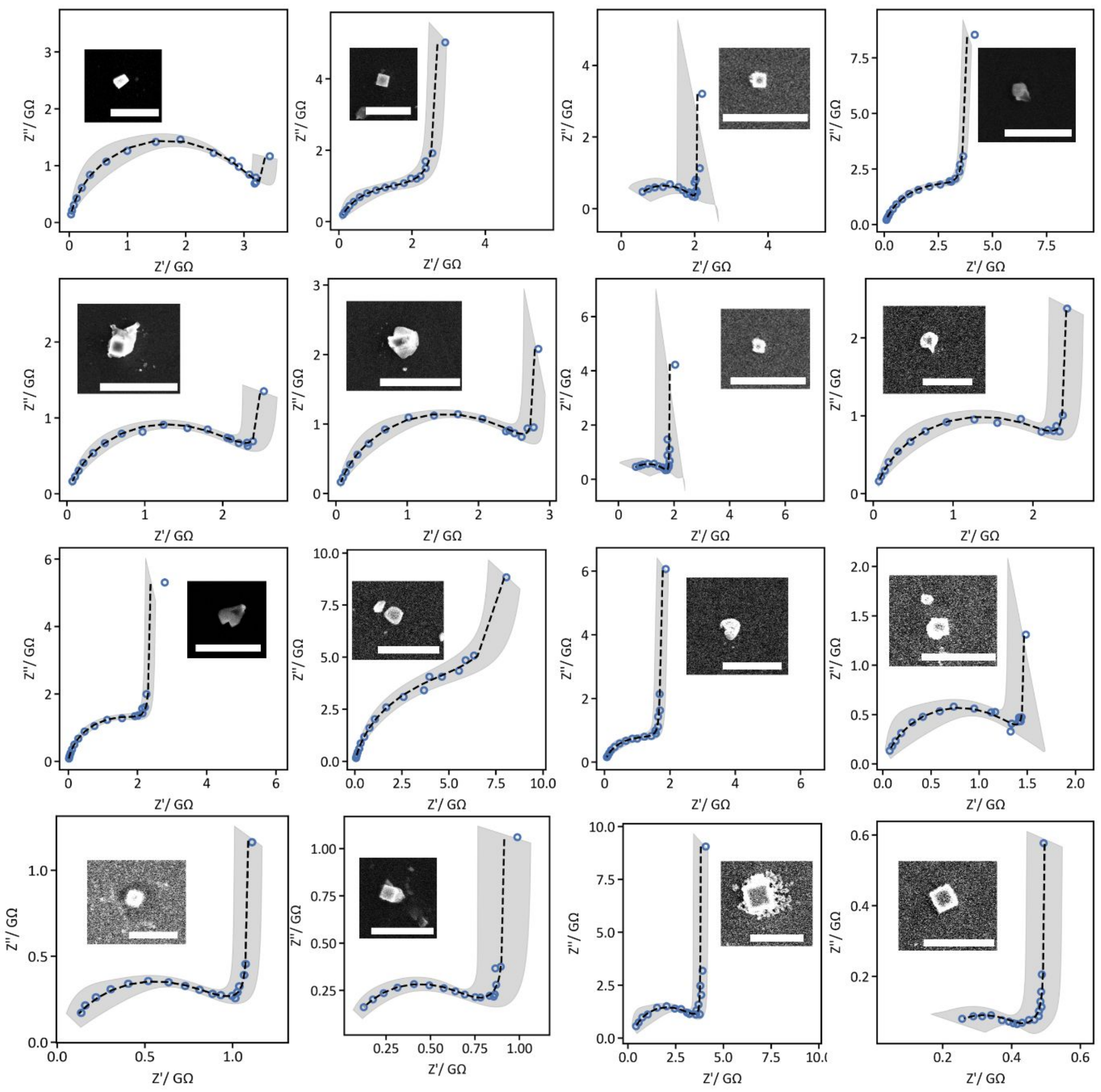

Figure S4. Impedance spectra (open circles), equivalent circuit fits (dashed curves, shaded regions represent 95% confidence intervals), and SEM images (insets) of the 16 individual nanoparticles studies herein. All SEM scale bars are 2 µm.

Table S1. Fitted Equivalent Circuit Parameters

Side 1 Length (nm)	Side 2 Length (nm)	R_s (MΩ)	R_{ct} (GΩ)	Q_{dl} (pF $s^{\alpha-1}$)	α	R_d (GΩ)	C_d (pF)	D (m^2 s^{-1})	j_0 (A m^{-2})
650	650	23.82	0.97	5.47	0.77	0.742	133.91	1.34E-15	62.7
330	330	189.17	1.94	2.02	0.74	0.059	48.43	4.66E-14	121.5
320	320	216.22	1.67	1.93	0.75	0.115	36.10	3.21E-14	150.4
900	900	205.09	3.30	0.72	0.88	2.578	17.03	3.04E-15	9.6
800	800	172.19	0.27	10.54	0.73	0.336	271.47	1.46E-15	150.1
420	420	17.19	1.45	5.05	0.82	0.446	121.61	2.46E-15	100.4
450	450	13.43	1.63	3.90	0.84	1.973	23.48	2.88E-15	78.0
440	360	19.30	7.48	3.38	0.85	16.042	17.81	4.67E-16	21.7
450	450	14.38	2.44	4.01	0.83	0.887	66.43	2.26E-15	51.9
440	310	18.26	2.79	3.49	0.85	0.960	77.49	1.79E-15	67.5
420	420	8.64	2.33	4.22	0.82	1.461	127.99	7.13E-16	62.5
305	305	18.06	4.19	3.74	0.81	2.580	16.19	3.19E-15	66.0
440	440	19.00	0.77	4.96	0.78	0.830	150.61	1.07E-15	173.1
500	500	16.06	2.20	4.83	0.78	4.644	29.06	9.88E-16	46.6
540	380	6.65	3.19	2.13	0.92	1.354	159.00	6.19E-16	39.3
490	320	4.59	2.65	3.91	0.91	1.312	27.28	3.73E-15	61.7

Equivalent Circuit Elements versus Particle Size

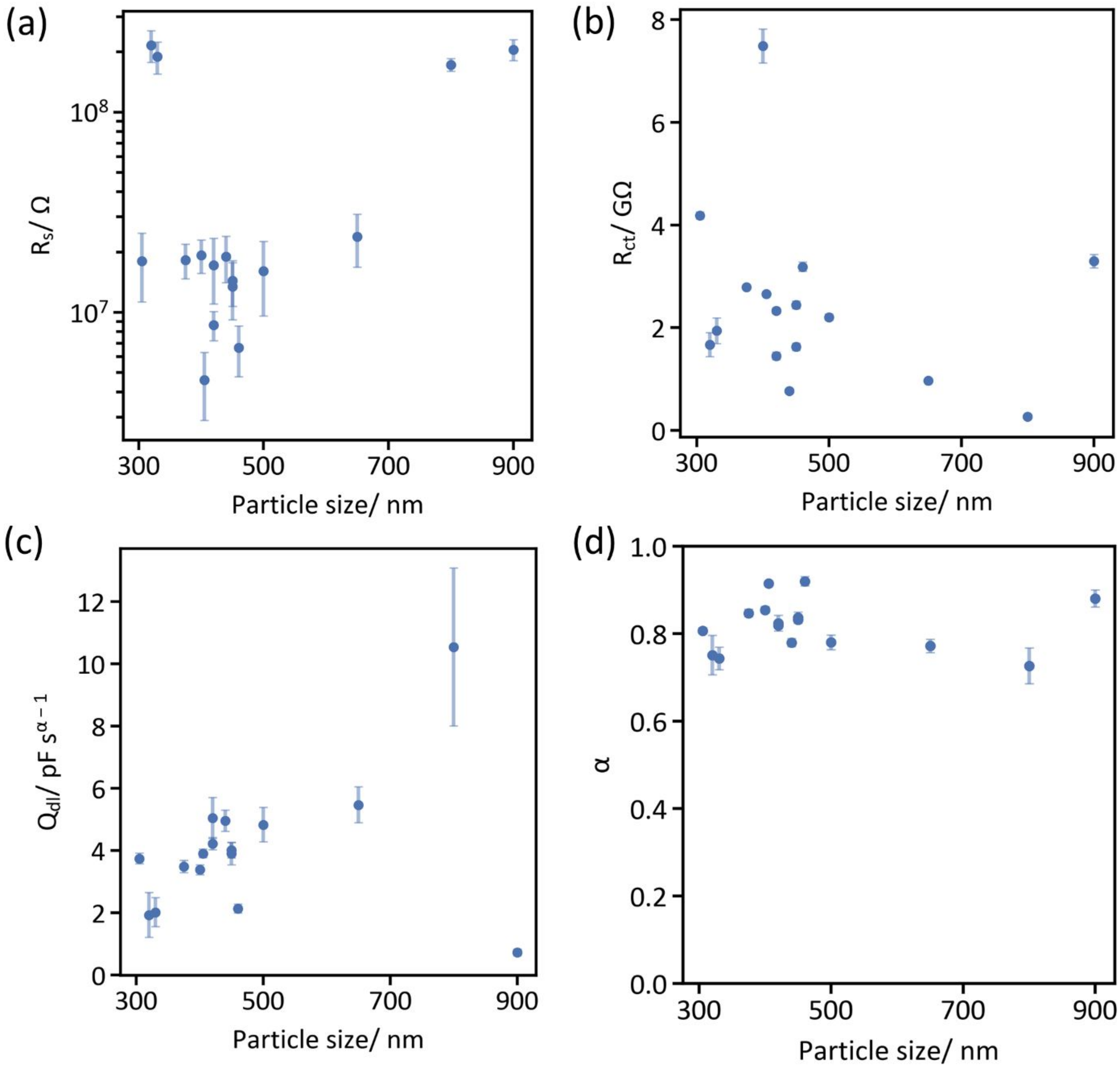

Figure S5. Equivalent circuit parameters plotted as a function of particle size. If a particle deviated from the cubic morphology, "particle size" refers to the average side length measured by SEM. (a) solution resistance, (b) charge-transfer resistance, (c) magnitude of double-layer capacitance constant phase element, and (d) CPE α parameter.

Q_{dl} (related to the magnitude of the double-layer capacitance) has a weak, positive correlation with particle size ($r = 0.31$ for all points; $r = 0.86$ excluding the 900 nm particle) likely caused by the double layers formed on the particles' surfaces contributing to the overall capacitance. Interestingly, R_s varies by almost two orders of magnitude. Some of this variation is likely caused by varying pipette orifice diameters and taper lengths, but some may be additionally caused by the location of the nanoparticle within the meniscus – a particle near the center of the droplet may block more ionic flux through the pipette than one near the edge.

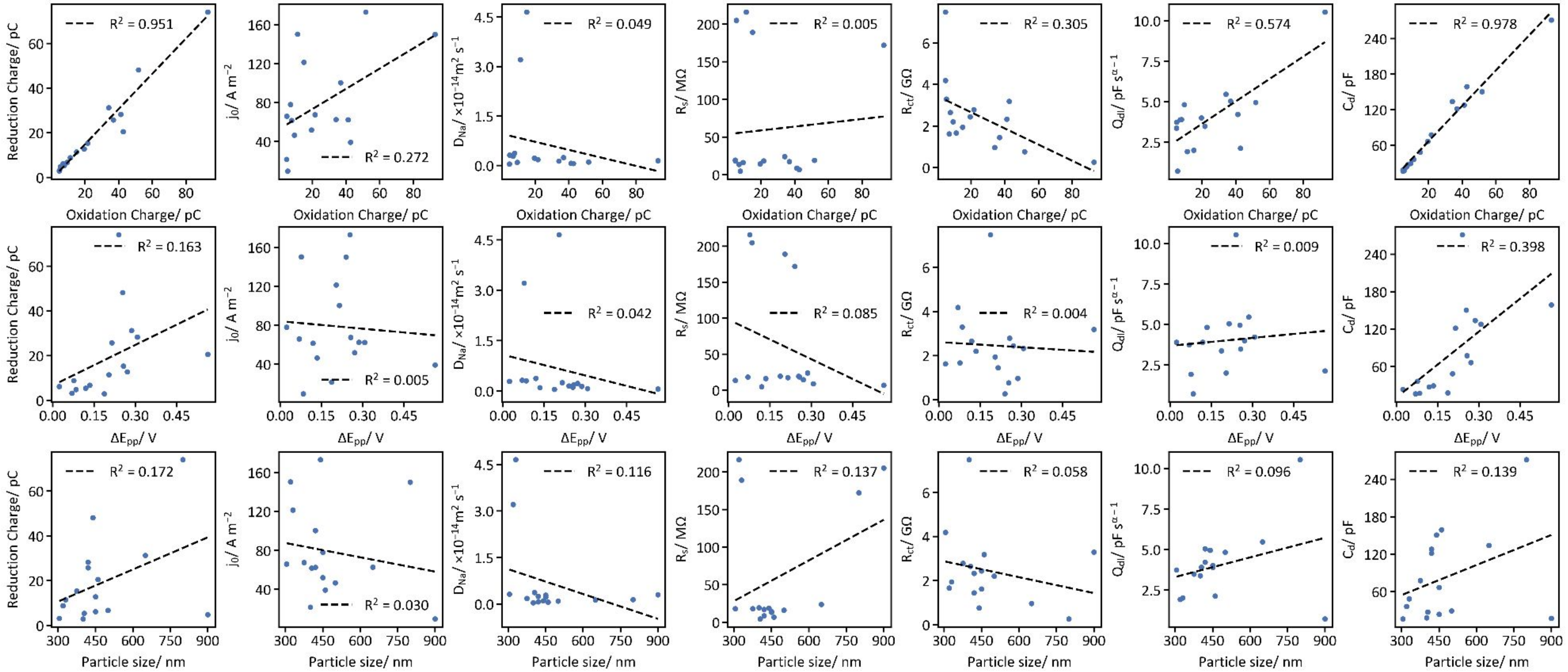

Figure S6. Correlation coefficients between various parameters extracted from single particle voltammograms, impedance spectra, and SEM. Oxidation charge: integrated charge from oxidative peak in CV. Reduction charge: integrated charge from reductive peak in CV. ΔE_{pp}: peak-to-peak separation extracted from CV. Particle size: particle side length measured by SEM. j_0: exchange current density. D_{Na}: sodium ion diffusion coefficient. R_s: solution resistance fitted from EIS. R_{ct}: charge transfer resistance fitted from EIS. Q_{dl}: magnitude of double layer capacitor element fitted from EIS. C_d: limiting low-frequency intercalation capacitance extracted from EIS diffusion model.

Most of these parameters (including those combinations not shown here) are not correlated to one another; here we discuss those that do show weak or strong correlations. The oxidation and reduction charge integrated from cyclic voltammograms are strongly related (R^2 = 0.951) to one another, as expected. Furthermore, they are strongly correlated (R^2 = 0.978) to the limiting, zero-frequency intercalation capacitance (C_d) extracted from the EIS diffusion model because both parameters are measures of the total (accessible) capacity of the particle. Interestingly, large C_d is also associated with larger peak-to-peak separations (ΔE_{pp}) in CV (R^2 = 0.398). This may be related to *iR* drop within the particle, as larger currents (larger charges and larger capacities) will cause larger *iR* drops and thus higher peak-to-peak separations.

Correlation between D_{Na} and j_0

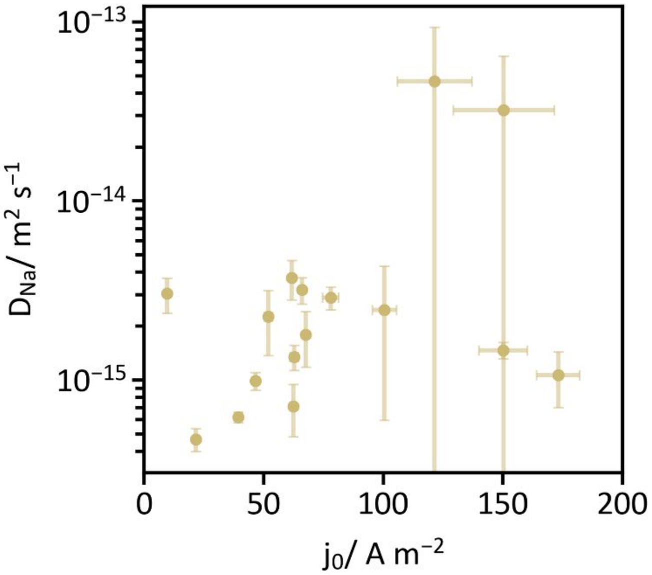

Figure S7. Relationship between D_{Na} and j_0 for the 16 particles studied herein. The two parameters are not correlated to one another.

Impedance Spectra at Varying AC Amplitudes

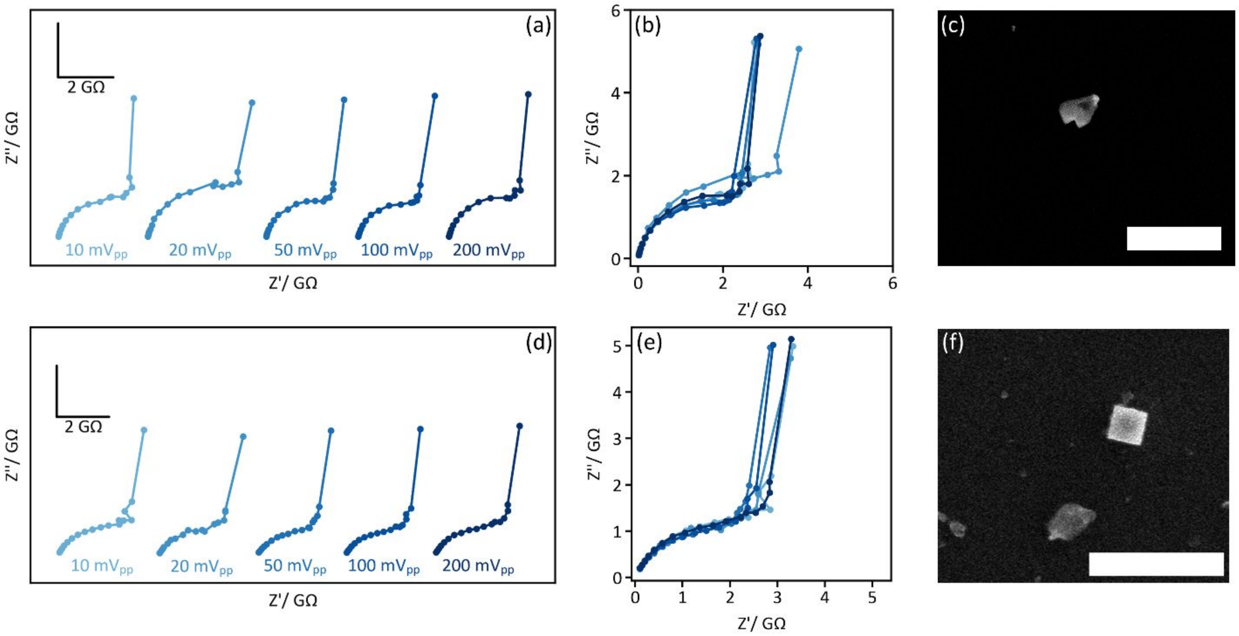

Figure S8. The measured impedance spectra are not significantly affected by the choice of AC amplitude in the range of 10 – 200 mV_{pp}. We measured impedance spectra on two particles with amplitudes of 10, 20, 50, 100, and 200 mV_{pp} (a, d). All five spectra overlap with one another (b, e) and do not display an obvious trend with AC amplitude or noticeable distortions at high amplitudes, either of which would suggest a non-linear response. The particles interrogated are shown in (c) and (f), scale bars are 2 μm.

Time-Resolved Impedance is Drift Free

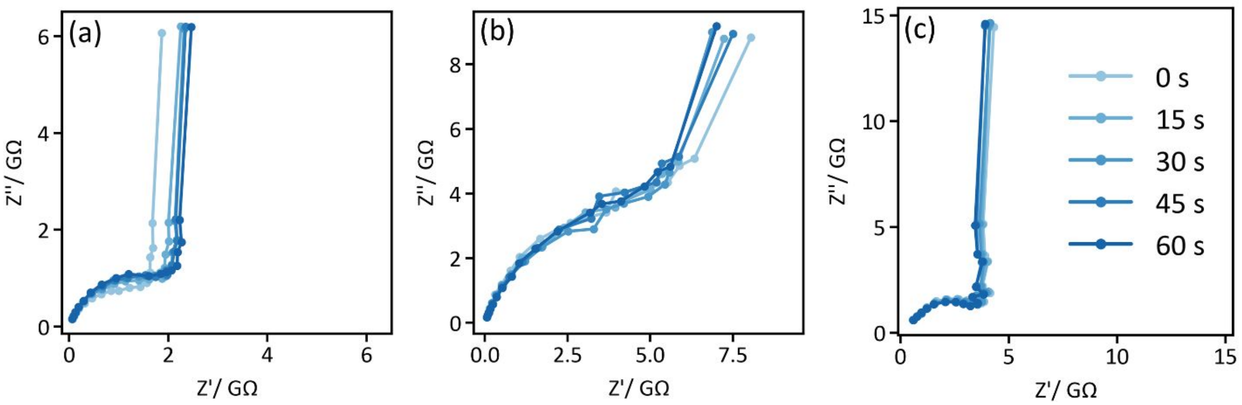

Figure S9. We measured impedance spectra every 15 s for a total of 60 s for three different particles. While the impedance of the first particle (a) drifted a small amount, the other two (b, c) were stable over the one-minute measurement. The drift visible in (a) is small compared to that observed in other particles (*vide infra*), which were excluded from analysis.

Example Particle with Fluctuating Conductivity

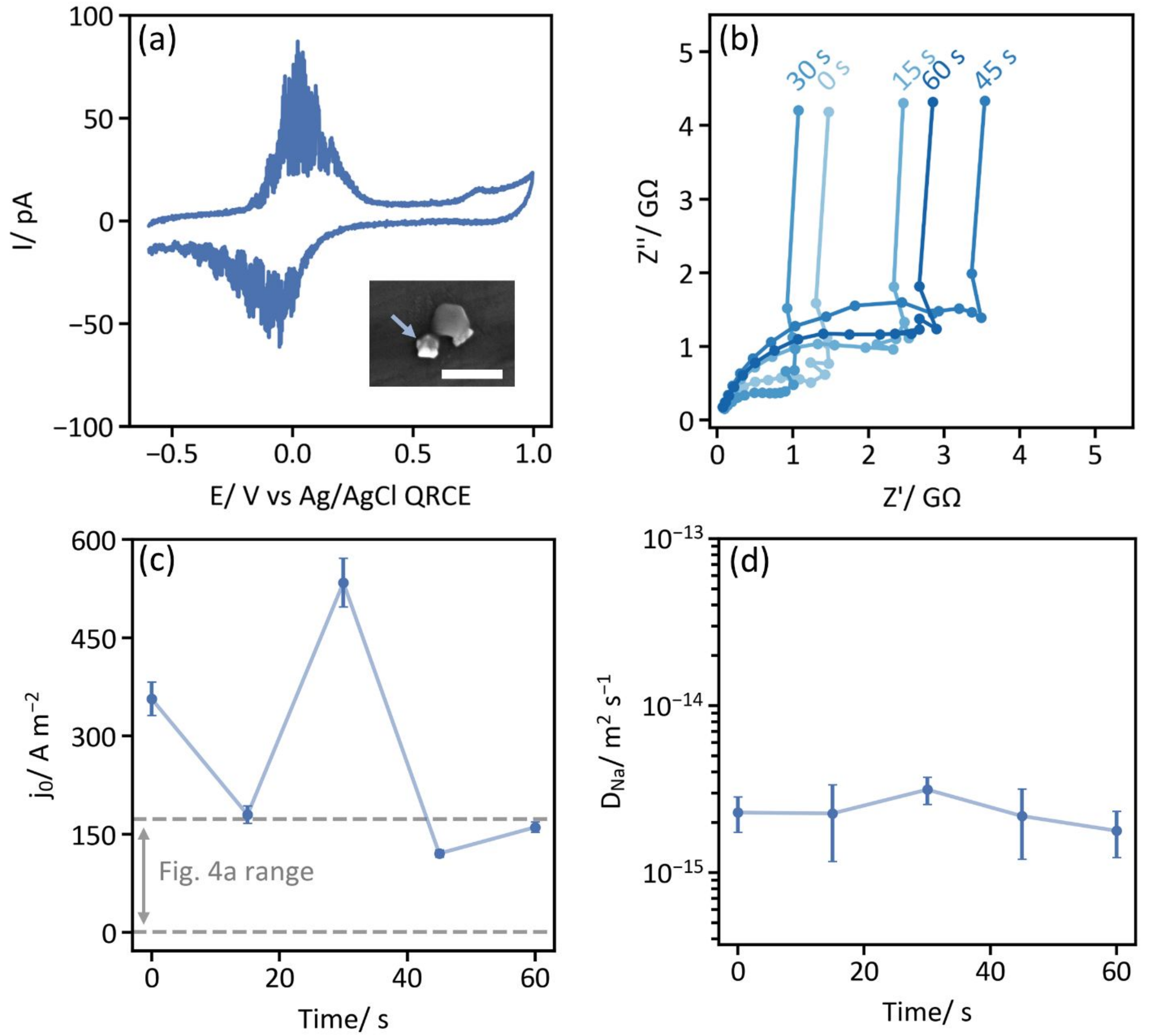

Figure S10. Cyclic voltammetry (a) and time-resolved impedance (b) of a PB nanoparticle which evidently had poor electrical contact with the glassy carbon substrate. Noisy spikes are visible in the voltammogram, while the impedance spectrum shifts sporadically in time. This is also visible in the fitted values of (c) j_0, which shift randomly across a wide range, while values of (d) D_{Na} remain relatively constant. Interestingly, the apparent values of j_0 for this small ($l \approx 240$ nm) nanoparticle are much higher than those shown in Figure 4a, the range of which is represented in (c). This particle and several others which showed similarly noisy voltammograms were excluded from our analysis. Error bars in (c) and (d) reflect the uncertainty in the equivalent circuit fit.

Impedance Spectrum with Poor Electrical Contact

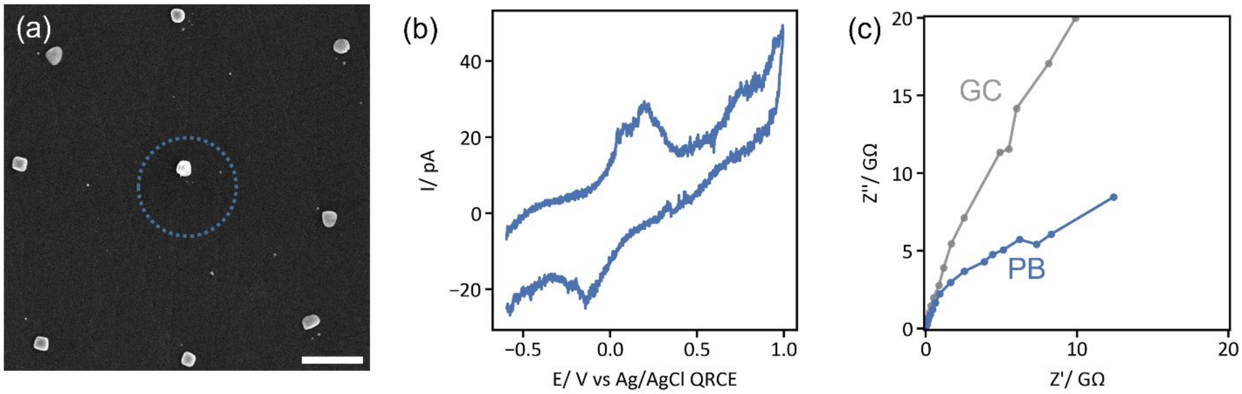

Figure S11. (a) SEM image (dotted circle represents the estimated SECCM droplet footprint), (b) cyclic voltammogram, and (c) impedance spectrum produced by a PB nanoparticle which apparently had poor electrical contact with the supporting glassy carbon electrode. It has a high impedance ($|Z| > 10$ GΩ) which is not distinguishable from the background at high frequencies. We attribute this to particularly poor electrical contact between the particle and the substrate, as was previously observed.[2] Between 30-50% of the individual particles we isolated displayed similar, extremely high impedance and were excluded from analysis.

References

(1) Popkirov, G. S.; Schindler, R. N. Optimization of the Perturbation Signal for Electrochemical Impedance Spectroscopy in the Time Domain. *Review of Scientific Instruments* **1993**, *64* (11), 3111–3115. https://doi.org/10.1063/1.1144316.

(2) Wei, W.; Yuan, T.; Jiang, W.; Gao, J.; Chen, H.; Wang, W. Accessing the Electrochemical Activity of Single Nanoparticles by Eliminating the Heterogeneous Electrical Contacts. **2020**. https://doi.org/10.1021/jacs.0c06171.
